# Melt-Processed Polybutylene-Succinate Biocomposites with Chitosan: Development and Characterization of Rheological, Thermal, Mechanical and Antimicrobial Properties

**DOI:** 10.3390/polym16192808

**Published:** 2024-10-03

**Authors:** Remo Merijs-Meri, Janis Zicans, Tatjana Ivanova, Linda Mezule, Aleksandrs Ivanickins, Ivan Bockovs, Juris Bitenieks, Rita Berzina, Alina Lebedeva

**Affiliations:** 1 Institute of Chemistry and Chemical Technology, Faculty of Natural Sciences and Technology, Riga Technical University, 1048 Riga, Latvia; janis.zicans@rtu.lv (J.Z.); tatjana.ivanova@rtu.lv (T.I.); ivans.bockovs@rtu.lv (I.B.); juris.bitenieks@rtu.lv (J.B.); rita.berzina@rtu.lv (R.B.); alina.lebedeva@rtu.lv (A.L.); 2 Water Systems and Biotechnology Institute, Faculty of Natural Sciences and Technology, Riga Technical University, 1048 Riga, Latvia; linda.mezule@rtu.lv (L.M.); aleksandrs.ivanickins@rtu.lv (A.I.)

**Keywords:** biocomposite, chitosan, melt compounding

## Abstract

The current research is devoted to the development and characterization of green antimicrobial polymer biocomposites for food packaging applications. The biocomposites were developed by melt compounding on the basis of two different succinate polymer matrices with varying chain stiffness—polybutylene succinate (PBS) or its copolymer with 20 mol.% of polybutylene adipate (PBSA). Fungi chitosan oligosaccharide (C98) and crustacean chitosan (C95) were used as antimicrobial additives. The rheological properties of the developed biocomposites were determined to clear out the most suitable temperature for melt processing. In addition, mechanical, thermal, barrier and antimicrobial properties of the developed biocomposites were determined. The results of the investigation revealed that PBSA composites with 7 wt% and 10 wt% of the C98 additive were more suitable for the development of green packaging films because of their higher ultimate elongation values, better damping properties as well as their superior anti-microbial behavior. However, due to the lower thermal stability of the C98 additive as well as PBSA, the melt processing temperatures of the composites desirably should not exceed 120 °C. Additionally, by considering decreased moisture vapor barrier properties, it is recommended to perform further modifications of the PBSA-C98 composites through an addition of a nanoclay additive due to its excellent barrier properties and thermal stability.

## 1. Introduction

The increasing concerns of food packaging waste on the environment are raising interest on more sustainable polymer materials and their composites to reduce environmental impact since a majority of the most widely used polymer materials for packaging are polyethylene (PE), polypropylene (PP), polyethylene terephthalate (PET) and polycarbonate (PC) [1]. For example, in 2021, each EU citizen generated 188 kg of packaging waste, of which 19% were related to polymers, demonstrating a ca 30% growth rate since 2010 [2].

To mitigate the environmental effect of plastic packaging, bio-based polymers could be considered a less harmful alternative to traditional plastics. Instead of petroleum-based polymers, they can be produced from renewable resources, e.g., biomass, thus reducing the dependency of fossil resources [3]. One of the viable solutions for bio-based packaging materials are biopolyesters due to their mechanical properties and processing properties [4]. Some of the examples of commercially marketed biopolyesters are polylactides (PLA), polybutylene succinate (PBS) and its copolymers, e.g., poly(butylene succinate-co-adipate) (PBSA), and polyhydrohyalkanoates (PHA), which have found a number of applications mainly in the biomedical field and packaging [5,6,7]. Already available biopolyesters are partially or completely produced from renewable biomass and are biodegradable in different environments with purposefully made properties for packaging applications [8]. Furthermore, in previous studies of biopolyester matrix composites with the aim of discovering a potential application in packaging film materials researchers have used various modifiers, mostly neat and organically treated clays [9,10,11,12,13], inorganic fillers, like zeolite [14], and biopolyester blends [15,16]. Particularly, to improve the antibacterial properties of biopolyester films, different additives are considered good candidates, for example, metal nanoparticles [17,18], like silver, copper, selenium, palladium and iron; metal oxide nanoparticles, like zinc oxide (ZnO), copper oxide (CuO) and magnesium oxide (MgO) [18,19]; carbon nanomaterials, like carbon nanotubes, fullerenes and graphene [18], as well as commonly used food preservatives, like chitosan, nanocellulose, curcumin, melanin and essential oils [18,20]. From these, chitosan proved to be a successful candidate in blends with a polymer matrix. Due to its polyelectrolyte nature, good film-forming properties and biocompatibility, chitosan shows a number of applications in drug delivery [21,22], edible coatings and dietary food supplements [23,24], environmental engineering [25,26] and elsewhere. The main advantages of chitosan over other antibacterial fillers are that it is derived from biological sources and it is biodegradable [27]. Presently, a number of different sources reported obtaining chitosan, mainly from crustaceans, insects and fungi [28], from which the latter source has considerable advantages with respect to a more sustainable production route. The reported antibacterial activity of chitosan, which could refer to packaging films, is mainly determined by its molecular weight (M_w_), deacetylation degree (DD) and its origin. Although the antimicrobial efficiency of chitosan differs by the type of specific bacteria, in general, it is commonly accepted that a low M_w_ chitosan has higher antimicrobial activity because of the ability to penetrate the cell wall [29,30]. Furthermore, usually a higher DD leads to the higher antibacterial activity of chitosan. The source of chitosan suggests that chitosan obtained from mushrooms has a higher antimicrobial activity than chitosan that originated from marine sources; however, this could be due to the fact that mushroom chitosan yields lower M_w_ [29,30,31]. Nevertheless, the inactivation mechanisms are still related to the ability of chitosan to attach to the cell walls of bacteria and fungi, their damage and subsequent leakage of intracellular components [29]. 

The reported antibacterial nature of chitosan could improve storage stability and increase the storage time of packaging films made from chitosan-modified polymers. Pure chitosan itself can be used as antibacterial film or as a coating directly on the surface of the food sample; however, chitosan has poor mechanical properties and moisture resistance [32]. Therefore, most studies are focused on chitosan blends with thermoplastic polymers by keeping the antimicrobial effect of chitosan and maintaining packaging film properties. Currently, most studies focus on low-density polyethylene (LDPE) blends with chitosan for uses in antibacterial packaging [33] with some potential uses for fish [34] and meat packaging [35]. The use of a LDPE matrix is beneficial because it is commonly used for food packaging; however, LDPE is produced from fossil resources. Therefore, it is necessary to increase the application of bio-based polymers since they can provide similar properties for commonly used packaging materials. 

Recent attempts in the application of thermoplastic bio-based polymer films with chitosan as an antibacterial agent suggests various biopolyester matrices such as PLA blends with PBS [36], PBSA [37] and PHB [37,38]. The results of mechanical and rheological properties show potential that these composites can be processed with extrusion, compression molding and other methods used in packaging material production. However, some articles are focused more on mechanical properties or only antibacterial properties of the obtained chitosan polymer composites. Also, pure PBS matrix blends with chitosan have not been widely evaluated, especially the effect of the bio-based polymer macromolecular configuration as well as the effect of the chitosan origin, deacetylation degree and molecular weight on antibacterial, mechanical and structural properties.

In the present work, bio-based polyester PBS and its copolymer with adipate (PBSA) with different macromolecular configurations were blended with two different types of chitosan with different M_w_ and DDs at various weight concentrations. To assess the potential use of PBS or PBSA-based binary systems with the selected chitosan additive for the manufacturing of extrusion-blown food packaging films, the mechanical, rheological, barrier and antimicrobial properties of the developed composites were characterized depending on the features of the bio-based polyester and the chitosan additive. Based on the obtained results, the optimal composition of antimicrobial bio-based food packaging film from a PBSA or PBS composite can be adopted depending on the chitosan type and concentration.

## 2. Materials

Bio-based polyesters (poly butylene succinate, PBS, and poly butylene succinate-co-adipate with 20 mol% of butyl adipate units, PBSA) from Natureplast (Mondeville, France) were used as thermoplastic polymer matrices. They are food safe and suitable for processing into packaging materials. PBSA has a density of 1.24 g/cm^3^ and a melt flow index (MFI) of 5 g/10min., whereas PBS has a density of 1.26 g/cm^3^ and a melt flow index (MFI) of 4–6 g/10min.

Two food-grade chitosan additives with different DDs and M_w_ were obtained from ChitoLytic (NF, Toronto, ON, Canada). C98 is produced from the cell walls of mushrooms and is reported as a very low-molecular-weight chitosan oligosaccharide with a very high deacetylation degree (DD = 98.2%), whereas a crab shell C95 chitosan has a higher M_w_ and a somewhat lower deacetylation degree (DD = 95.6%).

### Bio-Based Polymer Composite Film Preparation

Before processing, the bio-based polyester granules were dried at 80 °C for 2 h and then mixed with chitosan or chitosan oligosaccharide powder at 3, 5, 7 and 10 wt%. Then, the compositions were processed with a Labtech Engineering Co. Ltd. (Samutprakarn, Thailand) two-roll mill (LRM-S-110/3E) at 140 °C for 5 min, cooled and cut into small pieces. Samples at various thickness for characterization were hot molded with a Labtech Engineering Co. Ltd. (Samutprakarn, Thailand) hydraulic press (LP-S-50/S.ASTM) at 140 °C with a 2 min compression and a 1 min cooling cycle. 

## 3. Testing Methods

Rheological measurements were performed by using an Anton Paar AG (Graz, Austria) rheometer (Smart Pave 102) equipped with parallel plate geometry and a plate diameter of 25 mm. Rheological measurements in an oscillation mode were performed at various temperatures (100 °C, 120 °C, 140 °C and 160 °C) over the frequency range of 0.1–628 rad/s in the linear viscoelastic region (at a 1% shear strain). Additionally, rotational viscosity measurements were performed over the shear rate rage 10^−2^–10^2^ s^−1^ at the same temperatures. The gap between the plates was set to 1 mm. The specimens with a diameter of 25 mm and a thickness of 1.1 mm were cut from the compression-molded sheets.

Tensile tests were performed by using a ZwickRoell GmbH & Co. KG (Ulm, Germany) universal testing machine BDO-FM 020TN according to the EN ISO 527 standard. Young’s modulus (E), tensile strength at yield and break (σ_Y_ and σ_B_, respectively) as well as the tensile strain at yield and break (ε_Y_ and ε_B_, respectively) were determined from the stress–strain curves. Five samples of each composite system were tested. 

Dynamic mechanical thermal properties in tensile mode were determined according to the EN ISO 6721–2 standard. The storage modulus (E′) and loss angle (tanδ) were measured using a Mettler-Toledo, LLC (Columbus, OH, USA) dynamic mechanical thermal analyzer DMA/SDTA861. Experiments were performed in the temperature range from −75 °C to +50 °C at 1 Hz frequency.

Thermodynamic behavior in an air environment was evaluated over the temperature range from −80 °C to +200 °C at a ramp rate of 10 °C/min by using a Mettler-Toledo, LLC Mettler Toledo (Columbus, OH, USA)differential scanning calorimeter DSC 3. The mass of the specimen was ca 10 mg.

The degree of crystallinity (*x*) of the obtained compositions was calculated using the following equation:

(1) $$x=\frac{△H_{C}}{△H_{m}^{o}\left(1-W\right)}\times 100$$

where ∆*H*_c_ is the measured enthalpy of melting the test specimen and $△H_{m}^{o}$ is the theoretical enthalpy of melting the 100 % crystalline polymer, whereas *W—*is the fraction of the polymer in the composition. The average melting enthalpy value of 100% crystalline PBS around 100 °C was reported to be 220 J/g [39], whereas the melting enthalpy of 100% crystalline polybutylene adipate proposed by Van Krevelen is 135 J/g [40]. Consequently, by considering that PBSA contained 20 mol% of butyl adipate units, one may obtain that according to the rule of mixtures, the melting enthalpy of the 100% crystalline PBSA is 203 J/g. 

The thermogravimetric analysis in an air environment was evaluated over the temperature range from +25 °C to +800 °C at a ramp rate of 10 °C/min by using a Mettler-Toledo, LLC Mettler Toledo (Columbus, OH, USA)thermogravimetric analyzer TGA1/SF. The mass of the specimen was ca 10 mg.

The Fourier Transformation Infra-Red (FT-IR) spectra were obtained by a Thermo Fisher Scientific (Waltham, MA, USA) Nicolet 6700 spectrometer using an Attenuated Total Reflectance (ATR) technique. All the spectra were recorded over the wavenumber range between 650 cm^−1^ and 4000 cm^−1^ with a resolution of 4 cm^−1^.

Moisture vapor sorption measurements were made using the sealed test dish method. Test specimens were obtained from compression-molded plates with a thickness of ca 0.2 mm. The test area subjected to moisture exchange was ca 2.46 × 10^−3^ m^2^. The 50 ± 3% humid state was ensured by a saturated sodium dichromate Na_2_Cr_2_O_7_ × 2 H_2_O solution, whereas the dry state was ensured by anhydrous calcium chloride CaCl_2_ pebbles. The sorption tests were performed until the mass change was equalized following the sorption curve. The test temperature in the laboratory was 23 ± 2 °C, but the relative moisture content –50 ± 5%. The diffusion coefficient of the moisture sorption process was calculated according to Fick’s law from the initial straight portion of the sorption kinetics isotherms. 

Antimicrobial properties were measured via a reduction of fecal indicator bacteria (*Escherichia coli* ATCC25922) and baker’s yeast (*Saccharomyces cerevisiae*, food isolate). In prior antimicrobial tests, the cultures were incubated in a Tryptone Soya broth (TSB, Oxoid Ltd., London, UK) at 37 °C for 24 h. Then, they were thrice washed with a 0.1% sterile peptone solution through centrifugation (2 min, 2000× *g*, MiniSpin Plus Eppendorf). After a final resuspension of the pellet, a stock solution of 2—4 ×10^6^ cells/mL in 0.1% of sterile peptone was prepared. Cell numbers were estimated with microscopy by staining with 10 μg mL^−1^ of DAPI (4′,6-diamidino-2-phenylindole, Merck, Berlin, Germany) for 5–10 min and visualizing with epifluorescence microscopy (Ex: 365/12; FT 395; Em: >397, Zeiss Axioscope 5, Berlin, Germany) by a counting of 20 random fields of view.

For antimicrobial efficacy testing, visibly clean 2 × 2 cm^2^ polymer composite film samples were placed in sterile Petri dishes and covered with 0.8 mL of freshly prepared cell stock solution to ensure the full coverage of the film and avoid any leakages into the Petri dish. All samples were incubated in room temperature for 24 h. The stock solution was retained as untreated control and incubated under the same conditions. To avoid sample drying, sterile towels were soaked in a 0.1% sterile peptone solution and inserted in the Petri dishes next to the films. Microbial samples from each film were collected after 0, 3, 6 and 24 h. Decimal dilutions were inoculated on a Tryptone Bile X-Glucuronide (TBX) medium (Oxoid Ltd., London, UK) or Yeast growth agar (3 g/L yeast extract; 2 g/L peptone; 1 g/L potassium dihydrogen phosphate (KH_2_PO_4_); 0.5 g/L magnesium sulfate (MgSO_4_); 10 g/L glucose; 15 g/L agar) for *E. coli* or *S. cerevisiae*, respectively, and incubated at 37 °C for 48 h. Each sample was plated in duplicates. The results were expressed as colony-forming units (CFU) per milliliter. The detection limit was set to 0.33 CFU mL^−1^. Log reduction was calculated using the following equation log *reduction* = log (*N/N_0_*) where *N_0_* is the initial concentration of cultivable bacteria, and *N* is the concentration of bacteria after treatment.

## 4. Results and Discussion

### 4.1. Rheology

Melt rheology measurements were conducted over a certain temperature range to estimate the processing conditions of the obtained thermoplastic compositions. Consequently, further rheological investigations in rotation and oscillation at 1% shear strain modes were performed within 100–160 °C for PBSA and 120–160 °C for PBS matrix composites in compliance with the melting behavior of both biopolymers (maximum melting temperature of PBS and PBSA are 117 °C and 89 °C, respectively). 

**Figure 1:**
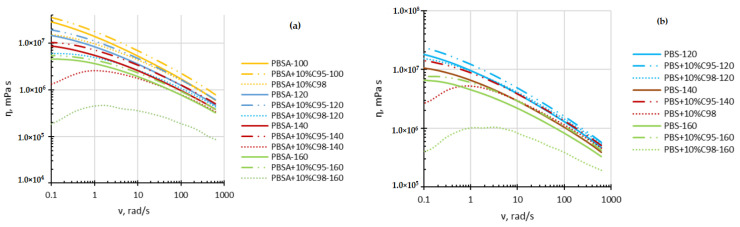
Dynamic viscosity *η* of PBSA (**a**) and PBS (**b**) composites with C98 and C95 chitosan additives as a function of frequency v at 100 °C, 120 °C, 140 °C and 160 °C.

Figure 1 demonstrates dynamic viscosity η of the investigated biopolyester composites with the used chitosan additives as a function of frequency ν at various testing temperatures. As demonstrated in Figure 1, the chitosan C95 addition causes the viscosity of the developed composites to increase, which is consistent with the addition of a rigid filler. In contrast, the chitosan C98 causes the viscosity of the developed composites to decrease even at the lowest testing temperature (100 °C), which denotes a high shear sensitivity of the low-molecular-mass chitosan oligosaccharide close to the melting temperature of the polymer matrix crystalline phase. By increasing the test temperature, the difference between the viscosity curve of the neat PBSA reference and the PBSA composite with 10 wt% of chitosan C98 proceeds. The viscosity curves of the other investigated composites lay between those of PBSA and PBSA + 10%C98 and are not shown here for better distinguishing the overall trend. It is important to mention that at 140 °C and beyond, viscosity curves of the C98 chitosan composites demonstrate the maxima point, denoting the beginning of thermo-oxidative degradation of the additive under the effect of an elevated temperature and prolonged testing time, while the frequency scan test was started from the highest frequency. Similar conclusions may be made from the PBS composites’ viscosity curves. If at 120 °C, the viscosity curve of a PBSA + 10 wt% composite do not differ much from that of the neat biopolymer matrix, then at 140 °C and 160 °C, the maxima in the PBSA + 10 wt%C98 composite’s viscosity curves are observed to be accompanied with a rapid drop of the viscosity curve values. 

**Figure 2:**
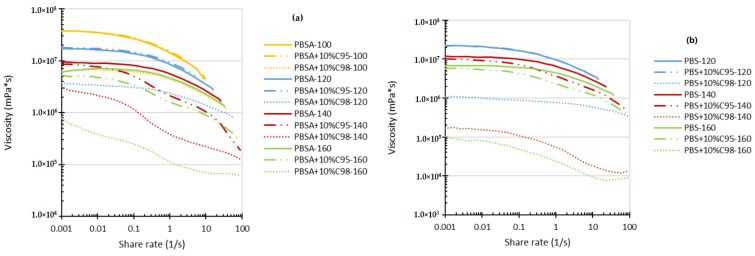
Shear viscosity of PBSA (**a**) and PBS (**b**) composites with C98 and C95 chitosan additives as a function of shear rate at 100 °C, 120 °C, 140 °C and 160 °C.

Additionally, rotational tests of the developed composites were performed at higher shear strain levels, In Figure 2, approaching the conditions characteristic of extrusion, blow-molding and thermoforming. In general, similar trends may be observed from the obtained viscosity–shear rate relationships *η*(*γ*). At the lowest testing temperature (100 °C), there is no great difference between the viscosity curves of the PBSA and the PBSA composite with 10 wt% of C95, whereas the viscosity curve of the PBSA+10 wt%C98 composite is ca one order of magnitude lower. Increasing the testing temperature up to 120 °C does not considerably change this pattern, i.e., the corresponding viscosity curves are smoothly shifted towards the direction of lower viscosity values. However, at 140 °C and 160 °C, the change of the *η*(*γ*) relationships for PBSA-C98 composites occurs, revealing a sigmoidal type of viscosity relationship, which may be related to the heterogeneity of the developed composites and the fact that at higher temperatures, the continuity of the biopolymer system is more easily disrupted, causing the chitosan additive to separate under rotation. Disregarding this, the rheological behavior of the developed composites may be described by power law within the certain shear rate range. From the viscosity curves displayed, it is evident that by increasing both the chitosan content and especially the temperature pseudoplasticity of the systems increases. From the viscosity relationships, one may also conclude that PBSA composites (especially those with C98) have increased shear sensitivity of PBSA contains composites, especially with C98. 

Trends in rheological behavior of PBS-based composites are rather similar, compared to those for PBSA composites with C98 and C95. Interestingly, by increasing temperature, the difference between *η*(*γ*) relationships for the PBS-based systems with C98 is larger in comparison to PBSA + C98. This, however, may be explained by a lower interfacial interaction at the melt state of the chitosan oligosaccharide within the rigid and bulky PBS in comparison to the more flexible PBSA.

### 4.2. Tensile Properties

**Figure 3:**
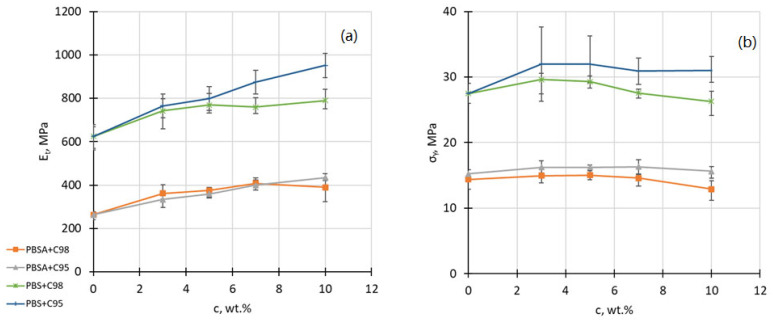

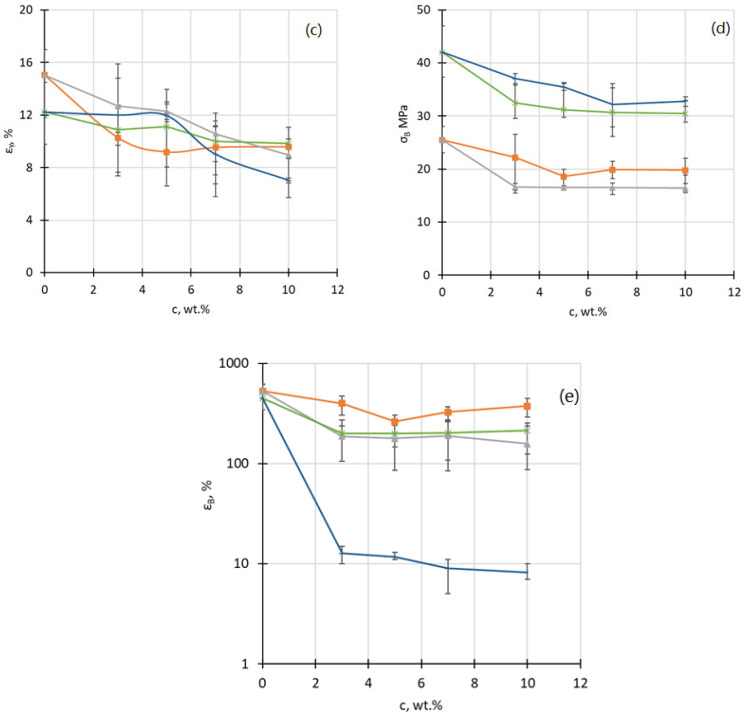
Tensile modulus *E_t_* (**a**), yield strength *σ_Y_* (**b**), strain at yield *ε_Y_* (**c**), ultimate strength *σ_B_* (**d**) and strain at break *ε_B_* (**e**) of PBS and PBSA composites with C98 and C95 chitosan additives as a function of the filler content.

In Figure 3a–e, the compositional relationships of tensile modulus *E_t_*, yield strength *σ_Y_*, yield strain *ε_Y_*, strength at break *σ_B_* and strain at break *ε_B_* and the chitosan additive content *c* of the developed PBS and PBSA composites are demonstrated. As already expected, the addition of rigid chitosan filler causes the elastic modulus of the composites to increase, demonstrating non-linear behavior with a gradual saturation at higher chitosan concentrations. In general, higher modulus values (by the factor 2–2.5) are demonstrated by the systems based on PBS due to a higher rigidity of its macromolecular chain, whereas a somewhat larger total increment of the modulus value is observed in the case of C95-containing systems, most probably due to its higher molecular mass. Thus, at a 10% concentration of C95 in PBS, the modulus increased by ca 50% from 624 MPa to 951 MPa. 

The influence of the chitosan addition on the yield strength of the developed PBS and PBSA composites is much smaller. However, a small increment of yield strength *σ_Y_* may be observed, especially for higher-molecular-weight chitosan C95-modified PBS composites (even up to 16% for the composite with 3 wt% of chitosan). At higher chitosan concentrations, *σ_Y_* reaches the saturation point and starts to decline due to the constrained deformability of the polymer matrix. In the case of C95-modified PBS composites, test tensile specimens are ruptured soon after reaching the yielding point. Concomitant yield strain *ε_Y_* decreases by an increasing concentration of the chitosan in the composites. Opposite to the *σ_Y_*_,_ the effect of the chitosan on *ε_Y_* is larger, even reaching a 43% decrement in the case of C95-modified PBS composites at a 10% concentration. Evidently, the effect of a higher-molecular-weight chitosan on deformation behavior is much larger than in the case of the chitosan oligosaccharide.

As already expected, the addition of chitosan to the polymer matrix caused a decrement of its ultimate stress *σ_B_* and ultimate strain *ε_B_* values. A somewhat higher decrement of *σ_B_* is observed for C95-modified PBS composites at the highest concentration level (above 40% from 52 MPa to 33MPa) in comparison to C98-reinforced PBSA composites showing a ca two times smaller decrement (from 25 MPa to 20 MPa). The influence of the chitosan additive on *ε_B_* values is even more dramatic, especially in the case of C95 -modified PBS composites, demonstrating a ca 50-fold decrement (from 450% to 8%). In contrast, the PBSA composite with a 10 wt% of C98 shows only a 1.4-fold decrement. 

In summary, one may conclude that PBSA composites with C98 demonstrate a more suitable mechanical property envelope for the expected extruded film blowing in comparison to other investigated composites.

### 4.3. Dynamic Mechanical Analysis

**Figure 4:**
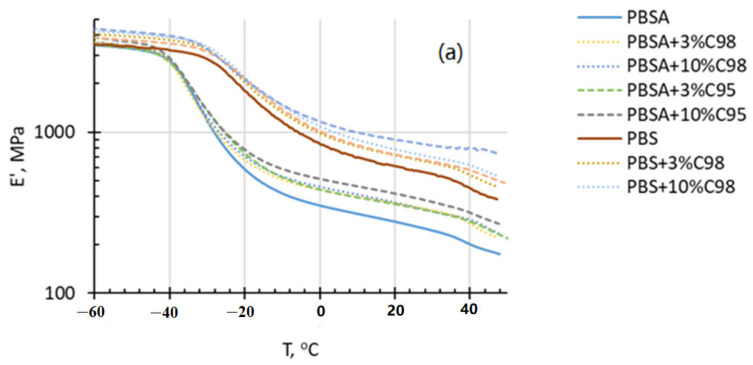

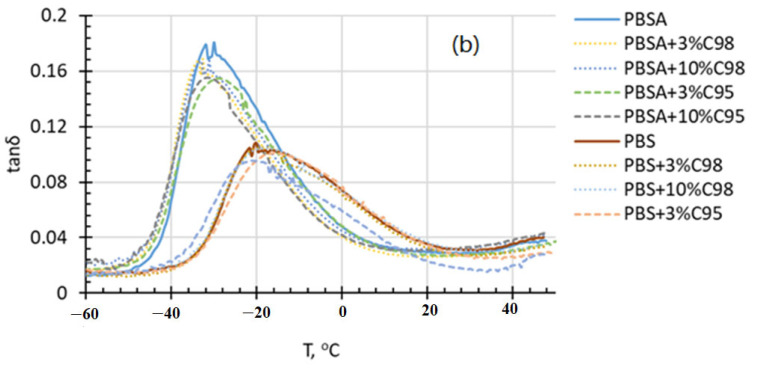
Storage modulus *E’* (**a**) and loss tangent *tanδ* (**b**) of PBS and PBSA composites with C98 and C95 chitosan additives as a function of temperature. −40.

Figure 4 demonstrates viscoelastic behavior (storage modulus *E’* and loss tangent *tanδ* temperature *T* relationships) of the developed PBS and PBSA composites over the broad temperature range. In compliance with the tensile test results, chitosan, especially higher-molecular-weight C95, positively contributes to the stiffness of the composites, revealing the modulus increase below and above the glass transition region of the respective polymer matrix. It is also clearly visible that the chitosan addition promoted the shift of the dynamic modulus–temperature *E(T)* relationships towards the direction of higher temperatures, especially for PBS and C95-containing composites revealing a stiffening effect of the rigid chitosan filler on the polymer matrix. Increments of the modulus decrease over the ca 30 °C may be associated with the relaxation of the so-called rigid amorphous phase. Apart from this, the observed main modulus decrease within the temperature region between ca −50 °C and ca 0 °C for PBSA-based systems, as well as between ca −30 °C and +20 °C for PBS-based systems, is associated with the glass transition of the polymer matrix phase.

The glass transition region of the investigated composites may be more exactly distinguished from the loss tangent–temperature relationships *tanδ(T)*, revealing an onset of the glass transition region at approximately −43 °C and −34 °C for neat PBSA and PBS, which complies rather well with the stated glass transition temperatures of these biopolymers [41]. As it is clearly seen, by increasing the chitosan additive content, the onset of glass transition peak of the composites gradually shifts to the direction of lower temperatures. Additionally, the peak values of the *tanδ(T)* relationships of the composites in general decrease with the chitosan additive content, thus denoting a reduced internal friction of the composites because of a weaker interaction between the polymer matrix and the filler. It may be seen that the effect of the chitosan additives on the damping behavior of the PBSA-based composites is larger in the case of the systems with C95, which in general complies with the more rapid drop of ultimate elongation values by increasing the chitosan additive content. Comparatively, the damping ability of PBSA-based composite systems is better, as in the case of the PBS-based counterparts. Furthermore, in the case of PBS composites with C95, *tanδ* peak shifts towards the direction of lower temperatures, which may be associated with decreased compatibility, complying well with the low ultimate tensile elongation values of the composite. 

### 4.4. Differential Scanning Calorimetry

The Main calorimetric parameters collected from the temperature scans of the investigated composites are summarized in Table 1 and Table 2. PBS and PBSA both are semi-crystalline polymers, demonstrating relaxations in their amorphous and crystalline phases, as it may be observed during the DSC scan. Consequently, the amorphous phase of neat PBS and PBSA was characterized by a glass transition temperature *T_g_* observed at −35 °C and −49 °C, respectively, matching well with the onset temperature of the *tanδ* peak recorded by DMTA. The crystalline phase of both the matrix polymers was characterized by overlapping melting/crystallization relaxations over the very broad temperature range of 25–138 °C. Such multi-peak thermal behavior of succinate polymers was already observed before, allowing us to distinguish four melting peaks, which are related to the presence of the so-called rigid amorphous phase and different distributions of lamellae having different thermal stability and a recrystallization on heating [42]. Chitosan and chitosan oligosaccharide additives in its turn were characterized with a broad endothermic peak and maxima at 105 °C for C95 and 107 °C for C98, respectively. This endothermic peak with maxima around 100 °C is characteristic for chitosan biopolymers and is commonly attributed to the moisture release [43]. In comparison to C98, C95 demonstrates a smoother and narrower endothermic peak because of its higher molecular mass. In the case of C98, the endothermic peak is likely overlapped with the beginning of a thermo-oxidative decomposition of the biopolymer close to 200 °C. Consequently, the influence of the chitosan additive on the properties of PBSA and PBS matrices is considerably different. In the case of PBSA + C98 composites, the introduction of C98 promotes a separation of the melting peaks by influencing the crystallization process of PBSA. As a result, the first and the second local maxima of the 1st melting peak *T_m1.1_* and *T_m_*_1.2_ decrease in the presence of the chitosan additive, denoting a decreased quality of the crystalline moieties of PBSA. Additionally, an approaching of the local melting maxima *T_m_*_1.1_ and *T_m_*_1.2_ is observed along with the rising chitosan oligosaccharide concentration. However, the total crystallinity degree of PBSA *X_t_* remains practically unchanged by the addition of C98. Concomitant, the position of the main melting maxima around 87–88 °C as well as the *T_g_* of the PBSA composites are insignificantly influenced (ca 1–3 °C) by the addition of C98. In the case of PBSA + C95 composites, the addition of the chitosan additive has not considerably influenced the multipeak crystallization behavior of the composite; however, an approaching of *T_m_*_1.1_ and *T_m_*_1.2_ has occurred, predominantly on the account of the increment of *T_m_*_1.1_. Concomitant, *X_t_* is increased by the addition of C95. Similar as in the case of PBSA + C98, *T_g_* of PBSA is not considerably affected. 

The influence of C98 on *T_g_* of PBS is also relatively small. In contrast to PBSA-based composites, only one melting maxima may be clearly distinguished up to ca 70 °C. This melting maxima, observed at 49 °C for the neat polymer matrix, decreases by increasing the chitosan content. Interestingly, C98-modified PBS composites demonstrate a cold crystallization peak, which is decreased along with an introduction of the chitosan oligosaccharide additive in the PBS matrix from 94 °C to 90–91 °C. The concomitant main melting peak is hardly affected by increasing the chitosan concentration. This denotes the reduced ability of PBS to crystallize in the presence of C98. In the case of the addition of C95 chitosan, the *T_g_* of the polymer matrix was influenced to the largest extent (decrement by 4 °C), which denotes the comparatively highest incompatibility of this composite, which also complies with the results of the tensile tests. Concomitantly, no cold crystallization is observed in the case of PBS + C95 composites. Evidently, C95 is more efficient in promoting crystallization of the polymer matrix. Consequently, the maxima of the 2nd melting peak *T_m2_* is increased for PBS + C95 composites from 94 °C (neat polymer matrix) to 102 °C. Nevertheless, similar to the case of the PBSA composite with C95, multipeak melting endotherms of PBS-based systems with C95 are observed. 

### 4.5. Thermogravimetric Analysis

**Figure 5:**
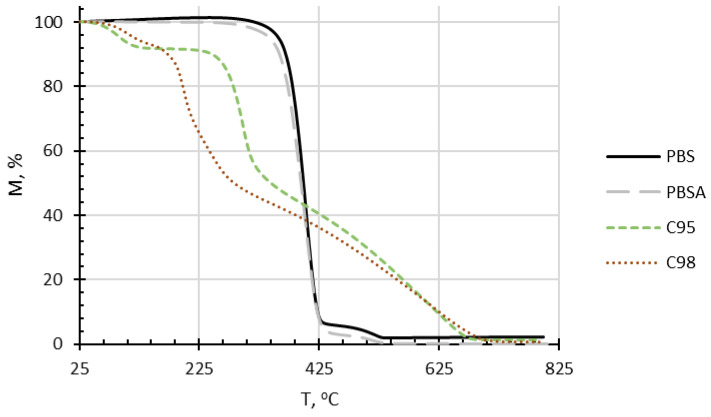
Thermogravimetric curves of neat PBS and PBSA matrices, as well as C95 and C98 chitosan additives.

**Figure 6:**
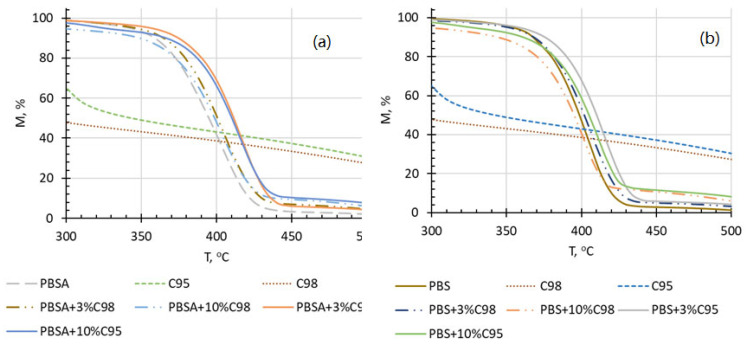
Thermogravimetric curves of PBSA (**a**) and PBS (**b**) composites with C95 and C98 chitosan additives as a function of temperature.

The thermooxidative stability of the composites primarily depends on the constituents’ chemical structure. As depicted in Figure 5 and Figure 6, PBS and PBSA degraded in two stages, whereas C95 and C98-in three stages. For chitosan, the first step in the weight loss curve is commonly related to the evaporation of water. In the case of C98, the first stage is broader than C95, and this is probably most connected with the beginning of thermooxidative decomposition of the chitosan oligosaccharide, as it was also observed in DSC thermograms. C95 has higher molecular mass and its thermooxidative decomposition starts after ca 225 °C. The 2nd weight loss stage of chitosan has been related with the dehydration of saccharide rings, as well as the depolymerization and decomposition of the acetylated and deacetylated units of the polymer, thus also depending on the degree of deacetylation of the biopolymer [44]. Consequently, C98 oligosaccharide with lower molecular weight demonstrated a more prominent 2nd weight loss stage in comparison to C95. The 3rd weight loss stage of both biopolymers is associated with further degradation of the thermooxidation products of stage 1 and stage 2. In comparison to the chitosan additives, PBSA and PBS demonstrate considerable higher thermooxidative stability, resulting in the beginning of rapid weight loss beyond ca 300 °C. Comparatively, the thermogravimetric curve of PBSA is slightly shifted to the direction of lower temperatures as well as results in a lower residual mass. This is evidently related to a lower thermal stability of the incorporated adipate units in comparison to succinate.

Taking into account the above mentioned, an introduction of the chitosan additive leads to a reduction in the onset decomposition temperature of the composite, especially at the highest chitosan additive concentration (10 wt%). Interestingly, a 3 wt% addition of the chitosan does not considerably influence the onset of thermooxidative decomposition on the investigated composites. Especially interesting is that the addition of the chitosan additive, predominantly C95, shifts the thermogravimetric curves in the range of the main decomposition stage to the direction of higher temperatures. Evidently, the chitosan additive positively contributes to the development of the gas-impermeable barrier layer during decomposition of the composites. This is also confirmed by residual mass levels of the developed composites, which at 500 °C have reached ca 7% at the highest chitosan additive concentration, independently of its type. The use of chitosan as a carbon and nitrogen source in the design of low-environmental-impact flame-retardant formulations has also been recently reported [45]. In general, the thermooxidative curves of the PBS-based composites are slightly shifted to the direction of higher temperatures in comparison to PBSA-based systems. “Derivative weight-Temperature” relationship curves of the developed composites are provided in the Figure S1.

### 4.6. Fourier Transform Infrared Spectroscopy

**Figure 7:**
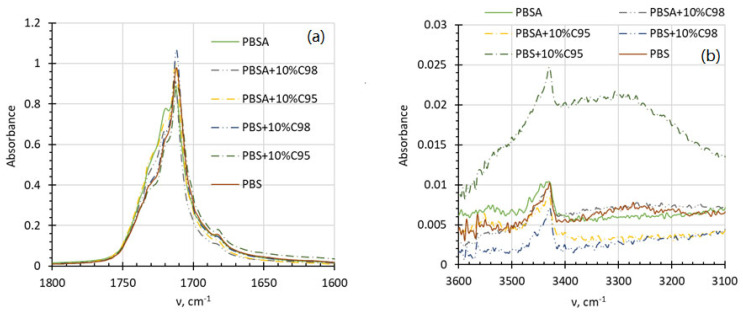
FTIR spectra of PBSA and PBS composites with C95 and C98 chitosan additives at carboxyl (**a**) and hydroxyl (**b**) absorption ranges.

FTIR spectra of the developed composites were measured in order to assess a potential interaction between the chitosan additive and the polymer matrix used. In addition, the FTIR measurements could allow us to follow up the possible chemical structure changes as a result of thermal decomposition of the constituents during the composite preparation and test specimen manufacturing. The corresponding FTIR spectra of the PBS- and PBSA-based composites in carboxyl and hydroxyl group absorption regions are summarized in Figure 7. From the composite FTIR spectra, one may conclude that characteristic peaks of the succinate biopolymer matrices are dominating, whereas the chitosan additive due to its small concentration (not exceeding 10 wt%) causes only intensity changes and shifts in the IR absorbance peaks of the composites. There are slight changes in the spectra of the PBS- and the PBSA-based composites, mainly detected in the wavenumber range between 1600–1880 cm^−1^ (Figure 7a), being in compliance with the observation made by Debuissy et al. [41] stating that the carbonyl stretching ester vibration slightly shifts to the direction of higher wavenumber values as the number of butylene-adipate groups is increased in the copolymer. In addition, in the presence of the C95 chitosan, an increment in the hydroxyl absorption range (3100–3600 cm^−1^) is observed, which may denote H-bonding. Although it is known that the used biopolymers are sensitive to thermal degradation, no signs of their considerable decomposition could be observed from the FTIR spectra (e.g., disappearance of hydroxyl band (dehydration), changes in the intensity of carbonyl band (thermal oxidation), diminution of the bands at 1660 cm^−1^, 1560 cm^−1^ (deacetylation). Full FTIR spectra of the investigated composites are provided in the Figure S2.

### 4.7. Antimicrobial Properties

**Figure 8:**
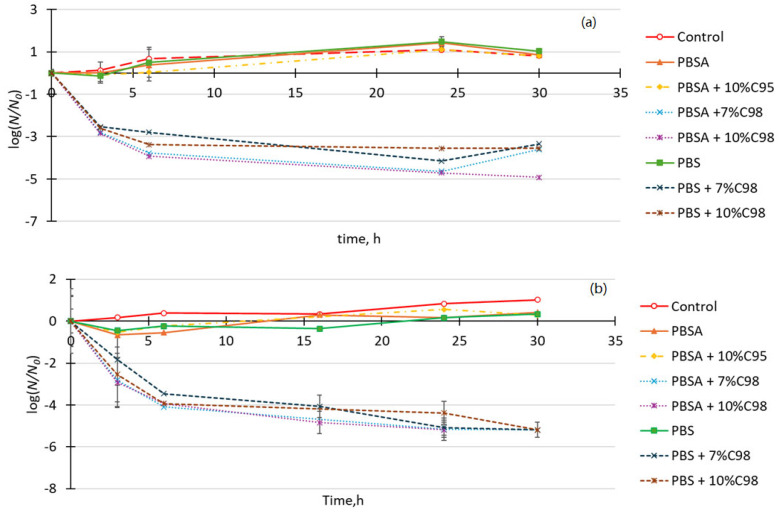
Antimicrobial efficacy of the developed composites on *E. coli* (**a**) and *S. cerevisiae* (**b**) cells in surface tests and low soiling conditions. The results are expressed as log CFU per ml. Each point represents at least 3 experimental repeats.

The antimicrobial properties of the developed composites were tested on two microorganisms—Gram-negative bacterium *Escherichia coli* that is representative of fecal pollution and yeast *Saccharomyces cerevisiae*—an indicator of spoilage in food industry. Antimicrobial efficacy of the developed composites on the cells of the mentioned test organisms is characterized in Figure 8. PBSA and PBS alone did not have any impact on cell viability—no significant changes in CFU counts were observed during 30 h of cell incubation on film surfaces. A similar trend of stable CFU counts (*p* > 0.05) was observed for PBSA with 10% C95 (Figure 8). Preliminary tests with the crustacean origin chitosan (C95) showed no antimicrobial effect, irrespective of the used biopolymer or chitosan concentration.

A supply of PBSA or PBS with 7 or 10% of fungal origin chitosan oligosaccharide (C98) resulted in a sharp decrease in CFU for both *E. coli* and *S. cerevisiae* in the first 6 h of treatment. A maximum reduction of 4.7 log *E. coli* CFU was obtained after a 24 h incubation on PBSA with 10% C98 (Figure 8a). The reduction in C98 to 7% in the composites resulted in only a minor decrease in efficacy (4.7 vs. 4.6 log after 24 h); however, a potential regrowth or resuscitation after 30 h of incubation was observed in these samples. The same trend was observed irrespective of the used polymer matrices. The observed phenomenon should be investigated further, if the polymer materials are intended for long-term storage. 

In comparison to *E. coli*, *S. cerevisiae* was more susceptible to PBS and > 5 log reduction was observed for both polymer matrix materials, irrespective of the used C98 concentration (Figure 8b). Prolonged incubation had no subsequent increase in *S. cerevisiae* CFU counts, indicating still unfavorable conditions after 30 h. 

### 4.8. Moisture Vapor Sorption

**Figure 9:**
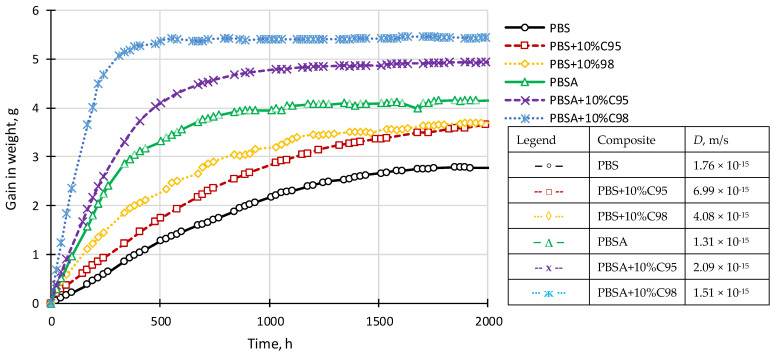
Moisture vapor sorption kinetic curves and diffusion coefficient *D* values of PBSA and PBS composites with C95 and C98 chitosan additives.

By considering potential applications of the developed composites for food packaging, water vapor permeability characteristics were analyzed. The water vapor sorption kinetic curves of the neat polymer matrices and its composites with the highest chitosan filler content are displayed in Figure 9. In general, the PBSA-based composites demonstrate higher water vapor sorption in comparison to PBS-based counterparts, which is explained by a more rigid macromolecular structure of the latter, as well as its denser crystalline structure. Furthermore, the influence of the C98 chitosan on the sorption behavior of the polymer is higher because of its larger water solubility in comparison to C95. Consequently, in the presence of the chitosan additive, the water vapor diffusion coefficient *D* increases as depicted in Figure 9. By considering the fact that increased moisture vapor diffusion may be disadvantageous for some packaging applications, further modification of the PBS and PBSA composites with organically modified nanoclay is expected.

## 5. Conclusions 

In the current research, the rheological, mechanical, thermal and sorption properties of a spectrum of polybutylene succinate (PBS) and polybutylene succinate-co-adipate copolymer (PBSA) compositions with two distinct antimicrobial chitosan additives (at 3, 5, 7, 10 wt% each), differing in its origin, chemical structure and molecular weight, (namely, fungi chitosan oligosaccharide C98 and crustacean chitosan C95), were obtained by melt processing and investigated for potential applications in sustainable food packaging. It was determined that the melt processing temperature of the developed composites should not exceed 120 °C, especially in the case of the most sensitive system based on PBSA and 10 wt% of C98. The replacement of the biopolymer matrix with PBS and the chitosan additive with C95 makes the systems more resistant to thermal processing, as also evidenced by thermogravimetric investigations under an oxidative environment. Stiffness and strength of the PBS and C95-containing systems are higher in comparison to those based on PBSA and C98, especially above the glass transition temperature of the polymer matrices occurring around −35 °C and −49 °C, respectively. In contrast, the PBSA composites with C98 oligosaccharide demonstrated the highest deformability, followed by PBS+C98, PBSA+C95 and PBS+C95, the latter demonstrating the largest brittleness at the maximum investigated chitosan additive content (10 wt%). In the meantime, only the composites with a 7 wt% and 10 wt% of C98 demonstrated a remarkable antimicrobial behavior of >4 log reduction for both *E. coli* and *S. cerevisiae* after 16 or 24 h of incubation in low soiling conditions, respectively, whereas the C95 chitosan additive appeared to be ineffective. Macromolecular configuration of the biopolymer had no significant impact on C98 efficacy. Consequently, the PBSA composites with 7 and 10 wt% of the fungal C98 additive have been suggested for potential application in thermoplastically processable sustainable antimicrobial packaging by blown film extrusion. Taking into the account the decrement of moisture vapor barrier properties of the obtained composites in the presence of the chitosan additive, it is expected to co-modify the composites with nanoclay and assess their antimicrobial effect on food-spoilage-related microorganisms, e.g., molds. Finally, it is expected to demonstrate the suitability of the developed binary and hybrid PBSA composites for blown film extrusion and packaging of certain food products.

## Figures and Tables

**Table 1:** Melting characteristics of the PBSA composites with the chitosan C95 and C98 additives.

Concentration of The Chitosan Additive	Glass Transition Temperature	1st Local Maxima of the 1st Melting Peak	2nd Local Maxima of the 1st Melting Peak	Crystallinity Degree of the 1st Melting Peak	Maxima of the 2nd Melting Peak	Crystallinity Degree of the 2nd Melting Peak	Total Crystallinity Degree
C %	*T_g_* °C	*T_m_*_1.1_ °C	*T_m_*_1.2_ °C	*X*_1.2_ %	*T_m_*_2_ °C	*X*_2_ %	*X_t_* %
PBSA + C98							
0	−50	50	67	One Multi maxima Peak Observed	87	One Multi maxima Peak Observed	26
3	−49	39	60	5	87	21	26
5	−47	41	61	5	88	20	25
7	−47	42	59	5	88	20	25
10	−47	39	59	5	88	22	27
PBSA + C95							
0	−50	50	67	One multi maxima peak observed	87	One multi maxima peak observed	26
3	−48	45	72		88		33
5	−48	44	71		87		27
7	−49	43	71		87		29
10	−49	44	71		87		29

**Table 2:** Melting characteristics of the PBS composites with the chitosan C95 and C98 additives.

Concentration of The Chitosan Additive	Glass Transition Temperature	Maxima of the 1st Melting Peak	Crystallinity Degree of the 1st Melting Peak	Maxima of the Cold Crystallization Peak	Crystallinity Degree of Cold Crystallization Peak	Maxima of the 2nd Melting Peak	Crystallinity Degree of the 2nd Melting Peak	Maxima of the 3rd Melting Peak	Crystallinity Degree of the 3rd Melting Peak	Initial Crystallinity Degree
C %	*T_g_* °C	*T_m_*_1_ °C	*X*_1_ %	*T_CC_* °C	*X_CC_*%	*T_m_*_2_ °C	*X*_2_ %	*T_m_*_3_ °C	*X*_3_ %	X_i_^*^%
PBS + C98										
0	−35	49	1	94	2	Not observed		117	31	31
3	−36	40	1	91	3	Not observed		115	30	30
5	−37	41	1	90	3	Not observed		115	33	30
7	−37	41	1	91	3	Not observed		115	32	30
10	−38	41	1	91	3	Not observed		115	33	30
PBS + C95										
0	−35	49	1	94	2	Not observed		117	31	31
3	−37	45	One multi maxima peak observed	No cold crystallization observed		102	One multi maxima peak observed	115	One multi maxima peak observed	41
5	−39	43				102		114		46
7	−39	45				101		114		45
10	−39	45				102		114		47

* Initial crystallinity degree *X_i_* was calculated by subtracting cold crystallization enthalpy values from the melting enthalpy values of the compositions and from the obtained enthalpy value calculating the crystallinity degree according to the Equation (1).

## Data Availability

The raw data supporting the conclusions of this article will be made available by the authors on request.

## Supplementary Materials

The following supporting information can be downloaded at: https://www.mdpi.com/article/10.3390/polym16192808/s1, Figure S1: Derivative weight-Temperature relationship curves of the developed composites (a) PBS; (b) PBSA; (c) 1st derivative; Figure S2: Full FTIR spectra of the developed composites (a) PBSA; (b) PBS.

## References

[1] Tajeddin B., Arabkhedri M. (2020). Polymers and food packaging. Polymer Science and Innovative Applications.

[2] Eurostat Statistics Explained, Packaging Waste Statistics. https://ec.europa.eu/eurostat/statistics-explained/index.php?title=Packaging_waste_statistics.

[3] Santhosh Kumar B.M., Keerthana P., Ravi G., Pant K., Sudhakar M. (2022). Bio polymer based food packaging using composite materials. Mater. Today Proc..

[4] Zhang Q., Song M., Xu Y., Wang W., Wang Z., Zhang L. (2021). Bio-based polyesters: Recent progress and future prospects. Prog. Polym. Sci..

[5] Jo S.Y., Lim S.H., Lee J.Y., Son J., Choi J.I., Park S.J. (2024). Microbial production of poly(3-hydroxybutyrate-co-3-hydroxyvalerate), from lab to the shelf: A review. Int. J. Biol. Macromol..

[6] Barletta M., Aversa C., Ayyoob M., Gisario A., Hamad K., Mehrpouya M., Vahabi H. (2022). Poly(butylene succinate) (PBS): Materials, processing, and industrial applications. Prog. Polym. Sci..

[7] Shekhar N., Mondal A. (2024). Synthesis, properties, environmental degradation, processing, and applications of Polylactic Acid (PLA): An overview. Polym. Bull..

[8] Nature Plast Biopolyesters. https://natureplast.eu/en/matiere/biopolyesters/.

[9] Chiu F.-C. (2017). Halloysite nanotube- and organoclay-filled biodegradable poly(butylene succinate-co-adipate)/maleated polyethylene blendbased nanocomposites with enhanced rigidity. Compos. B Eng..

[10] Taleb K., Saidi-Besbes S., Pillin I., Grohens Y. (2022). Biodegradable Poly(Butylene Succinate) Nanocomposites Based on Dimeric Surfactant Organomodified Clays with Enhanced Water Vapor Barrier and Mechanical Properties. ACS Omega.

[11] Taleb K., Pillin I., Grohens Y., Saidi-Besbes S. (2021). Polylactic acid/Gemini surfactant modified clay bio-nanocomposites: Morphological, thermal, mechanical and barrier properties. Int. J. Biol. Macromol..

[12] Dang K.M., Yoksan R., Pollet E., Avérous L. (2020). Morphology and properties of thermoplastic starch blended with biodegradable polyester and filled with halloysite nanoclay. Carbohydr. Polym..

[13] Sathish Kumar R.K., Dhilipkumar T., Jessie J.A., Gaayathri K.K., Arumugam S. (2023). Advancements in bio-polymeric composite materials for active and intelligent food packaging: A comprehensive review. Mater. Today Proc..

[14] Threepopnatkul P., Preedanorawut R. (2022). Poly(lactic acid) and polybutylene succinate films incorporated with modified zeolite. Mat. Today Proc..

[15] Yang C., Tang H., Wang Y., Liu Y., Wang J., Shi W., Li L. (2019). Development of PLA-PBSA based biodegradable active film and its application to salmon slices. Food Packag. Shelf Life..

[16] Feijoo P., Mohanty A.K., Rodriguez-Uribe A., G´amez-P´erez J., Cabedo L., Misra M. (2023). Biodegradable blends from bacterial biopolyester PHBV and bio-based PBSA: Study of the effect of chain extender on the thermal, mechanical and morphological properties. Int. J. Biol. Macromol..

[17] Ding Y., Yu W., Zhang J., Liu W., Zhu F., Ye Y., Zheng Q. (2023). Enhanced antibacterial properties of poly(butylenes succinate-co-terephthalate)/Ag@MgO nanocomposite films for food packaging. Polym. Test..

[18] Priyadarshi R., Roy S., Ghosh T., Biswas D., Rhim J.-W. (2022). Antimicrobial nanofillers reinforced biopolymer composite films for active food packaging applications-A review. Sustain. Mater. Techno..

[19] Packialakshmi J.S., Kang J., Jayakumar A., Park S., Chang Y., Kim J.T. (2023). Insights into the antibacterial and antiviral mechanisms of metal oxide nanoparticles used in food packaging. Food Packag. Shelf Life.

[20] Wangprasertkul J., Siriwattanapong R., Harnkarnsujarit N. (2021). Antifungal packaging of sorbate and benzoate incorporated biodegradable films for fresh noodles. Food Control.

[21] Kaur M., Sharma A., Puri V., Aggarwal G., Maman P., Huanbutta K., Nagpal M., Sangnim T. (2023). Chitosan-Based Polymer Blends for Drug Delivery Systems. Polymers.

[22] Smagina V., Yudaev P., Kuskov A., Chistyakov E. (2023). Polymeric Gel Systems Cytotoxicity and Drug Release as Key Features for their Effective Application in Various Fields of Addressed Pharmaceuticals Delivery. Pharmaceutics.

[23] Silva I.M.V., Machado F., Moreno M.J., Nunes C., Coimbra M.A., Coreta-Gomes F. (2021). Polysaccharide Structures and Their Hypocholesterolemic Potential. Molecules.

[24] Fatima M., Mir S., Ali M., Hassan S., Khan Z.U.H., Waqar K. (2024). Synthesis and applications of chitosan derivatives in food preservation-A review. Eur. Polym. J..

[25] Maroulas K.N., Trikkaliotis D.G., Metaxa Z.S., AbdelAll N., Alodhayb A., Khouqeer G.A., Kyzas G.Z. (2023). Super-hydrophobic chitosan/graphene-based aerogels for oil absorption. J. Mol. Liq..

[26] Yudaev P., Semenova A., Chistyakov E. (2024). Gel based on modified chitosan for oil spill cleanup. J. Appl. Polym. Sci..

[27] Oladzadabbasabadi N., Mohammadi Nafchi A., Ariffin F., Jeevani Osadee Wijekoon M.M., Al-Hassan A.A., Dheyab M.A., Ghasemlou M. (2022). Recent advances in extraction, modification, and application of chitosan in packaging industry. Carbohydr. Polym..

[28] Lia J., Zhuang S. (2020). Antibacterial activity of chitosan and its derivatives and their interaction mechanism with bacteria: Current state and perspectives. Eur. Polym. J..

[29] Ke C.-L., Deng F.-S., Chuang C.-Y., Lin C.-H. (2021). Antimicrobial Actions and Applications of Chitosan. Polymers.

[30] Alimi B.A., Pathania S., Wilson J., Duffy B., Celayeta Frias J.M. (2023). Extraction, quantification, characterization, and application in food packaging of chitin and chitosan from mushrooms: A review. Int. J. Biol. Macromol..

[31] Wijesekara T., Xu B. (2024). New Insights into Sources, Bioavailability, Health-Promoting Effects, and Applications of Chitin and Chitosan. J. Agric. Food Chem..

[32] Sarfraz M.H., Hayat S., Siddique M.H., Aslam B., Ashraf A., Saqalein M., Khurshid M., Sarfraz M.F., Afzal M., Muzammil S. (2024). Chitosan based coatings and films: A perspective on antimicrobial, antioxidant, and intelligent food packaging. Prog. Org. Coat..

[33] Kusumastuti Y., Putri N.R.E., Timotius D., Syabani M.W. (2020). Rochmadi Effect of chitosan addition on the properties of low-density polyethylene blend as potential bioplastic. Heliyon.

[34] Reesha K.V., Panda S.K., Bindu J., Varghese T.O. (2015). Development and characterization of an LDPE/chitosan compositeantimicrobial film for chilled fish storage. Int. J. Biol. Macromol..

[35] Giannakas A.E., Salmas C.E., Leontiou A., Baikousi M., Moschovas D., Asimakopoulos G., Zafeiropoulos N.E., Avgeropoulos A. (2021). Synthesis of a Novel Chitosan/Basil Oil Blend and Development of Novel Low Density Poly Ethylene/Chitosan/Basil Oil Active Packaging Films Following a Melt-Extrusion Process for Enhancing Chicken Breast Fillets Shelf-Life. Molecules.

[36] Monika, Katiyar V., Mulchandani N. (2019). Generalized kinetics for thermal degradation and melt rheology for poly (lactic acid)/poly (butylene succinate)/functionalized chitosan based reactive nanobiocomposite. Int. J. Biol. Macromol..

[37] Van den Broek L.A.M., Knoop Rutger J.I., Kappen Frans H.J., Boeriu Carmen G. (2015). Chitosan films and blends for packaging material. Carbohyd. Polym..

[38] Ilyas R.A., Aisyah H.A., Nordin A.H., Ngadi N., Zuhri M.Y.M., Asyraf M.R.M., Sapuan S.M., Zainudin E.S., Sharma S., Abral H. (2022). Natural-Fiber-Reinforced Chitosan, Chitosan Blends and Their Nanocomposites for Various Advanced Applications. Polymers.

[39] Signori F., Pelagaggi M., Bronco S., Righetti M.C. (2012). Amorphous/crystal and polymer/filler interphases in biocomposites from poly(butylene succinate). Thermochim. Acta.

[40] Van Krevelen D.W. (1997). Chapter 5-Calorimetric Properties, Properties of Polymers.

[41] Debuissy T., Pollet E., Avérous L. (2017). Synthesis and characterization of biobased poly(butylene succinate-ran-butylene adipate). Analysis of the composition-dependent physicochemical properties. Eur. Polym. J..

[42] Wang X., Zhou J., Li L. (2007). Multiple melting behavior of poly(butylene succinate). Eur. Polym. J..

[43] Gill P., Moghadam T.T., Ranjbar B. (2010). Differential Scanning Calorimetry Techniques: Applications in Biology and Nanoscience. J. Biomol. Technol..

[44] Eulalio H.Y.C., Rodrigues J.F.B., Santos K.O., Peniche C., LiaFook M.V. (2019). Characterization and thermal properties of chitosan films prepared with different acid solvents. Rev. Cuba. Quím..

[45] Malucelli G. (2020). Flame-Retardant Systems Based on Chitosan and Its Derivatives: State of the Art and Perspectives. Molecules.

