# Supplementary material for: Melt-Processed Polybutylene-Succinate Biocomposites with Chitosan: Development and Characterization of Rheological, Thermal, Mechanical and Antimicrobial Properties
Source: Polymers (Basel). 2024 Oct 3;16(19):2808. doi: 10.3390/polym16192808 (PMC11478647; doi:10.3390/polym16192808)
Supplement: Supplementary file 1 [file polymers-16-02808-s001.zip › polymers-3203619-supplementary.pdf]

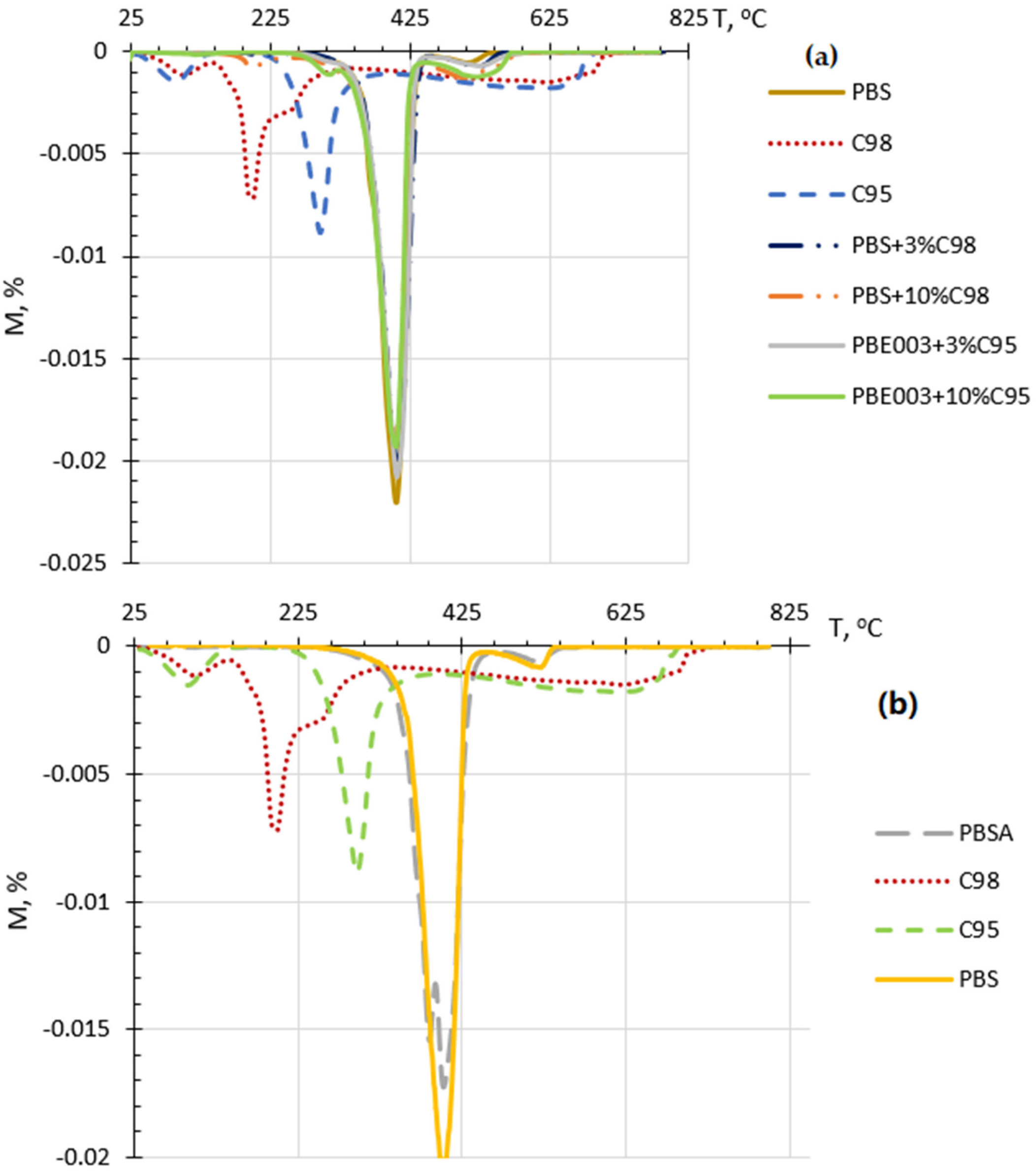

25
225
425
625
825 T, ºC
0
-0.005
-0.01
-0.015
-0.02
-0.025
M, %
(a)
PBS
C98
C95
PBS+3%C98
PBS+10%C98
PBE003+3%C95
PBE003+10%C95
25
225
425
625
825
T, ºC
0
-0.005
-0.01
-0.015
-0.02
M, %
(b)
PBSA
C98
C95
PBS

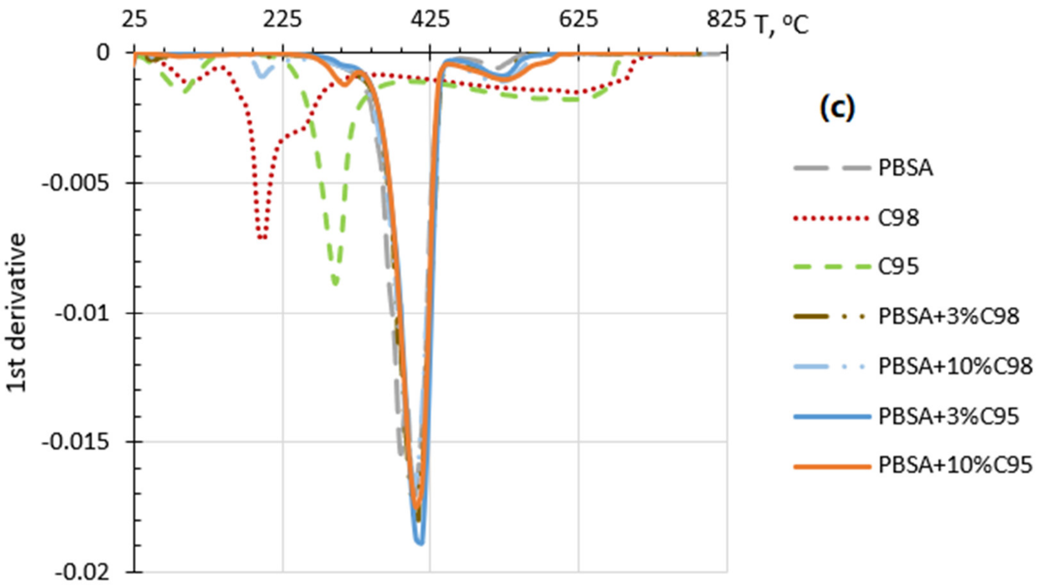


Figure S1: Derivative weight - Temperature" relationship curves of the developed composites (a) PBS; (b) PBSA; (c) 1st derivative

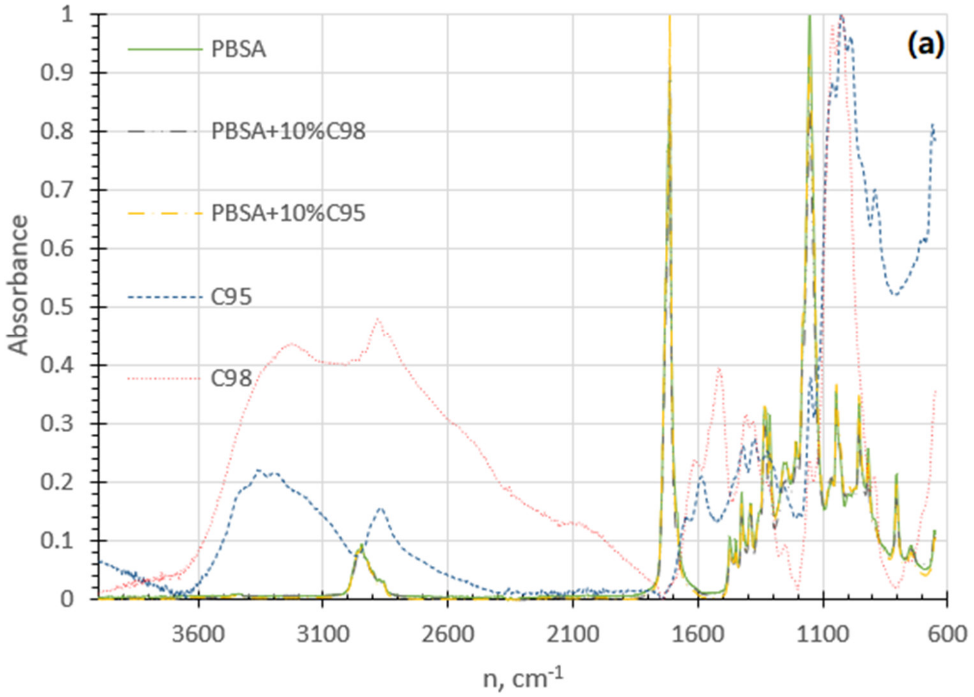

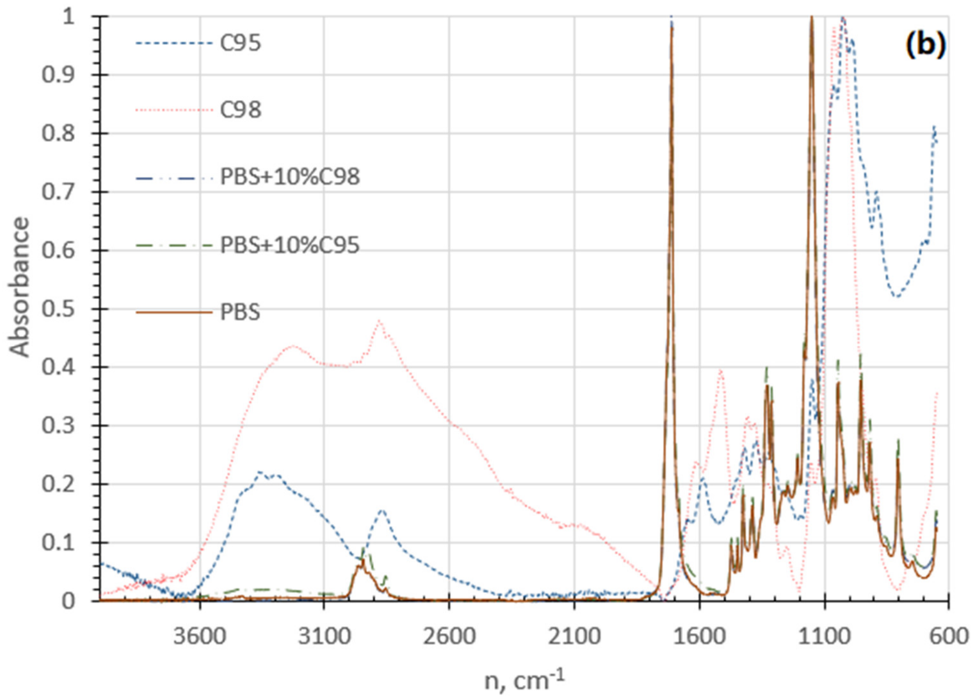


Figure S2: Full FTIR spectra of the developed composites (a) PBSA; (b) PBS
